# A simple method to maintain immobilization and elevation of the hand in infants and children

**DOI:** 10.4103/0970-0358.41130

**Published:** 2008

**Authors:** Siddharth K. Karanth, Mukund R. Thatte, Arvind M. Vartak

**Affiliations:** Department of Plastic Surgery and Burns, Bai Jerbai Wadia Hospital for Children, Parel, Mumbai, Maharashtra, India

Dear Sir,

Hand elevation and immobilization is an essential step following hand surgeries, hand trauma and in oedematous and inflammatory conditions of the hand. This is difficult to maintain in infants and young children (less than four years old) in whom hand elevation is required. The patients in this age group are restless and irritable in the postoperative period and are understandably uncooperative to the elevation and immobilization regimes at other times too. Usage of bandage slings and bolsters/pillows are common ways to immobilize the limb. In addition, the staff nurses and parents/attendants of the patients are advised to maintain a constant vigil in keeping the hand immobilized and elevated. Older children tend to be more cooperative and elevation and immobilization can be maintained by conventional methods.

**Figure 1:**
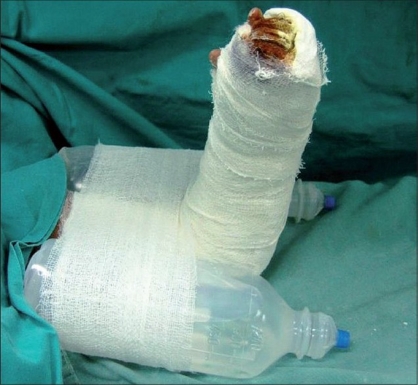
This is a postoperative image following a surgery of the hand in a child. An above-elbow slab was applied and two infusion bottles were fastened gently to the arm

We have devised a simple technique to overcome the aforementioned problems with the utilization of infusion bottles. Following the surgery and/or dressings, we routinely give an above-elbow slab with plaster of Paris, dorsally, maintaining the elbow at 90° flexion and the hand in a supine position. Two infusion bottles are placed on either side of the arm and wrapped with a bandage [Figure 1].

The right-angled upper elbow slab has two advantages: first, it doesn't slide down the limb as seen in the lower elbow slabs of the hand and secondly, it helps in maintaining limb elevation. An infusion bottle (500 ml crystalloid solution) is inexpensive and easily available in wards and operating rooms. When they are fastened as bolsters to the arm, they stabilize the limb from falling sideways and the combined weight of the infusion bottles and the slab makes it heavy for the infants and younger children to lift. This ensures immobilization of the limb and prevents them from thrashing the limb around. When the child is to be breastfed or to be taken in the parent's lap or carried around, only the bandage is unfastened and the infusion bottles removed. And when the child is put back on the bed, the bottles and the bandages can be reapplied easily by the parent(s) or the staff nurse.

This technique is simple, effective in maintaining position and immobilization and quickly executable at no extra cost to the patients.

